# Not all Perfectionists Are as They Are Assessed: An Investigation of the Psychometric Properties of the Perfectionism Inventory in the Teaching Profession

**DOI:** 10.3389/fpsyg.2021.624938

**Published:** 2021-02-10

**Authors:** Elena Mirela Samfira, Laurenţiu P. Maricuţoiu

**Affiliations:** ^1^Teacher Training Department, Banat University of Agricultural Sciences and Veterinary Medicine from Timisoara, Timisoara, Romania; ^2^Department of Psychology, West University of Timisoara, Timisoara, Romania; ^3^Clinica Universitara de Terapii si Consiliere PsihoPedagogica, West University of Timisoara, Timisoara, Romania

**Keywords:** perfectionism, The Perfectionism Inventory, schoolteacher, confirmatory factor analysis, internal validity

## Abstract

Perfectionism has been studied for almost 30 years. In the present study, we investigated the internal validity of The Perfectionism Inventory (PI—Hill et al., 2004) in an occupation that encourages perfectionistic tendencies in own behavior or in students' behavior. We collected data from a large sample of schoolteachers (*N* = 633, 81.18% female, 63.02% from urban areas, 46.66% from secondary schools, mean age = 42.11 years) recruited using a snowball sampling approach, and we analyzed the factor structure of the PI using confirmatory factor analyses. We found that the 8-factor structure of PI provided a reasonable fit root mean square error of approximation [RMSEA = 0.055, 90% CI = (0.053–0.057); SRMR = 0.071]. However, additional analyses revealed problematic divergent validity only in the case of the scales associated with self-evaluative perfectionism, not in the case of the scales associated with conscientious perfectionism. We found that teachers displayed distinguishably different forms of perfectionism only when it referred to own person, not when it referred to perfectionism imposed to others. Based on these findings, we suggested that the PI could provide a useful framework for investigating the role of conscientious-related forms of perfectionism in the development of teacher beliefs regarding their school behavior.

## Introduction

Perfectionism is a complex, multidimensional personality trait (Hill et al., 2016; Stoeber, 2017) which is strongly related to various affective disorders such as anxiety, depression (Egan et al., 2011), suicide tendencies (Smith et al., 2018), and insomnia (Schmidt et al., 2018). When they define perfectionism, scientists refer to the idea of having high standards of performance (Hewitt et al., 2017), and to the idea of having overly critical evaluations of own behavior (Frost et al., 1990; Hewitt and Flett, 1991).

In educational settings, perfectionism is an important research topic because it is related to achievement and because it is highly relevant for understanding goal attainment (Flett and Hewitt, 2016). The educational environment encourages high standards of academic achievement (Flett et al., 2009; Schruder et al., 2014), therefore it can encourage perfectionistic tendencies in students and in teachers (Gilman and Ashby, 2006). As part of the educational environment, teaching is a complex task that requires teachers to set achievement goals to students, and to monitor how students fulfill these goals (Shim et al., 2020). Despite its relevance for the outcomes of the educational environment (for a detailed discussion, see Starley, 2019), research on teacher perfectionism is limited (Starley, 2019; Shim et al., 2020). As recent research suggested that perfectionistic tendencies are associated with a wide range of affective disorders (Egan et al., 2011; Maricuţoiu et al., 2019), it is important to understand how perfectionistic tendencies are manifested by teachers in the educational environment.

In this paper, our aim was to investigate the internal validity of a comprehensive measure of perfectionism [i.e., the Perfectionism Inventory (PI)—Hill et al., 2004], on a large sample of schoolteachers. There are two main arguments for conducting this research. First, perfectionism is an important teacher variable that is associated with teaching efficacy and teacher burnout (Ghorbanzadeh and Rezaie, 2016), while teacher pressure to perform was found to be related to clinical symptoms in students (Lozano et al., 2019). This means that accurate assessment of teacher perfectionism can be important for understanding both teacher-related and student-related variables. Second, the teacher perfectionistic tendencies can be enhanced by the nature of their job. As teachers are required by their students and by their peers to behave without making mistakes (Pelletier et al., 2002), it is possible that they manifest different forms of perfectionism simultaneously. Therefore, the differential diagnosis of various forms of perfectionism might be difficult in the teachers' case.

Initial research studies identified the two forms of perfectionism: adaptive (or positive) and maladaptive (or negative) perfectionism (Terry-Short et al., 1995; Flett and Hewitt, 2006; Ulu and Tezer, 2010). Adaptive perfectionism is generally understood as perfectionistic strivings (i.e., putting effort into achieving high-quality outcomes and high performance standards), while maladaptive perfectionism is generally seen as having perfectionistic concerns (i.e., overcritical self-views, uncertainty and doubts regarding own capacities or regarding the outcomes of own actions). Although these forms of perfectionism seem to be functionally opposite, they are generally seen as independent forms of perfectionism that could be observed simultaneously in one's behavior (Stoeber et al., 2020). Beyond the functional vs. dysfunctional aspects attributed to perfectionism, the concept evolved toward a multidimensional approach in the 1990s. This means that researchers identified various forms of manifestation for perfectionistic strivings and for perfectionistic concerns, which were later seen as super-ordinate (or second-order) dimensions of perfectionism. Initially, two multidimensional perspectives of perfectionism dominated the literature (and the perspective developed by Frost et al., 1990; i.e., the perspective suggested by Hewitt and Flett, 1991). The perspective developed by Hewitt and Flett (1991) described perfectionism as a three-dimensional construct: self-oriented perfectionism, other-oriented perfectionism, and socially-prescribed perfectionism. *Self-oriented perfectionism* (*SOP*) reflects the tendency of an individual to set exacting standards for oneself and stringently evaluating and censuring own behavior. *Other-oriented perfectionism* (*OOP*) reflects the tendency of an individual to have exaggerated expectations about capabilities of others and to be overcritical with them. *Socially-prescribed perfectionism* (*SPP*) reflects the perceived need of an individual to attain high standards and expectations imposed by significant others, who exert pressure on them to be perfect (Hewitt and Flett, 1991). On the other hand, the perspective proposed by Frost et al. (1990) had six dimensions: concern over mistakes, personal standards, parental expectations, parental criticism, doubting of actions, and organization. *Concern over mistakes* was conceptualized as negative responses to mistakes, a tendency to interpret mistakes as failures, and a tendency to believe that an individual will lose the respects of others after failures. *Personal standards* was conceptualized as the settings of very high standards of performance and a tendency for self-evaluation based on performance. *Parental expectations* and *Parental criticism* reflects the tendency to believe that parents set excessive goals and are overly critical. *Doubting of actions* was conceptualized as a tendency to feel projects/results are not accomplished to satisfaction. *Organization* was conceptualized to stress the importance of neatness, organization, and order (Frost et al., 1990).

Individuals with perfectionistic strivings (i.e., adaptive perfectionists) tend to recognize their limitations and find appropriate coping strategies (Flett et al., 2009), see the difficulties they face in performing tasks as real challenges, and themselves as competent persons (Frost et al., 1990). Adaptive perfectionism is closely related to experiencing strong feelings of pride associated with low feelings of shame and guilt (Stoeber et al., 2008), and it was seen as a healthy form of perfectionism (Flett and Hewitt, 2006). By contrast, people with perfectionistic concerns (i.e., *maladaptive perfectionists*) put effort to be perfect, but see themselves as being too far from perfection (Slaney et al., 2002). Maladaptive perfectionists are more likely to think in a dichotomous manner, often being overwhelmed by fear of failure and not disappointing others (Gilman and Ashby, 2006). However, recent evidence (e.g., Maricuţoiu et al., 2019) suggested that extreme levels of adaptive perfectionism are also associated with clinical syndromes of depression and anxiety.

More recently, these perspectives were combined in a comprehensive questionnaire by Hill et al. (2004). *The Perfectionism Inventory* (PI—Hill et al., 2004) combined all dimensions theorized in the 1990s in a single questionnaire with eight scales. The main advantage of using the PI over the utilization of the existing scales was that it reduced the redundancy resulted from the overlapping concepts of these scales, while providing a comprehensive assessment of perfectionism (Hill et al., 2004). In the PI, the authors grouped perfectionism dimensions in two main categories: Conscientious Perfectionism (included the factors *Organization, High Standards for Others, Striving for Excellence*, and *Planfulness*), and Self-evaluative Perfectionism (included the factors *Concern over Mistakes, Need for Approval, Parental Pressure*, and *Rumination*). The existence of second-order factors was confirmed through a confirmatory analysis of the eight scale scores (Hill et al., 2004; Cruce et al., 2012). Hill et al. (2004) argued that the use of the eight facets of perfectionism (i.e., rather than the use of the two second-order factors) could provide a more psychologically meaningful image of perfectionism.

Given the multidimensional nature of perfectionism, researchers delimited between core facets of perfectionistic concerns and strivings, and variables that are peripheral to perfectionism (Stoeber and Otto, 2006; Stricker et al., 2019). The peripheral variables include antecedents of perfectionism development (i.e., parentally prescribed perfectionism), perfectionism oriented to others, and correlates of perfectionism (e.g., planfulness, rumination, or need for approval). Therefore, the PI (Hill et al., 2004) is a diagnostic tool that includes both core and peripheral perfectionism variables. Research studies that used the PI in work contexts reported that its scales are positively correlated with perceived stress and burnout (Craioveanu, 2014), or with active coping (Crăciun and Dudău, 2014). Both Conscientious perfectionism and Self-evaluative perfectionism were positively related to stress and burnout, and had similar correlation values with these scales (Craioveanu, 2014). However, high Conscientious perfectionism was more strongly associated with high levels of active coping, as compared with Self-evaluative perfectionism (Crăciun and Dudău, 2014). On the other hand Self-evaluative perfectionism displayed stronger negative relationships with both forms of social support coping, as compared with Conscientious perfectionism (Crăciun and Dudău, 2014). Finally, the overall score of the PI was strongly associated (i.e., correlations above.40) with most symptoms assessed by the Symptom Checklist (SCL-90-R; Derogatis, 1983), except for phobia and obsession (Craioveanu, 2014).

Although the idea of combining scales from different perspectives into a single inventory was commendable, a major limitation of the Hill et al. (2004) work was that they did not present evidence for the psychometric properties of the entire set of items. In their initial work, the authors of the PI conducted separate principal components analyses for each factor. However, a single factor analysis (confirmatory or exploratory) of the entire set of items is still missing from the literature. Therefore, because we still have little evidence to assess the internal validity of the PI scales, we aimed to fill this gap by conducting a thorough investigation of the PI psychometric properties.

Perfectionism was also studied in the educational context, where high standards are promoted. Fletcher et al. (2014) stated that perfectionism exists and develops within contexts that involve relationships with parents, teachers, colleagues, coaches, and other categories. School teachers are particularly prone to developing occupational stress (Stoeber and Rennert, 2008; Sadoughi, 2017; e.g., Salmela-Aro et al., 2019), and perfectionism plays an important role in this process (Flett et al., 1995; Friedman, 2000). The educational environment is a context in which high standards are encouraged (Flett et al., 2009) and performance is expected (Schruder et al., 2014). These aspects can enhance the students' and the teachers' perfectionist tendencies (Gilman and Ashby, 2006).

Lortie (1975) argued that teachers suffer from a culture of high standards, they frequently realize that they cannot live up to the standards imposed by themselves or by others. Schoolteachers perceive a real social pressure to be perfect—from students, peers, and parents (Pelletier et al., 2002), and the fear of imperfection determines teachers to be more authoritarian (Dinkmeyer et al., 1980). More recently, Shim et al. (2020) reported that teachers that are concerned regarding their mistakes are less likely to promote the intrinsic value of learning to their students. In a similar vein, high levels of perfectionism concerns are associated with teaching efficacy and teacher burnout (Ghorbanzadeh and Rezaie, 2016). To prevent such perfectionistic behaviors and their consequences, Jones (2016) suggested that highly experienced teachers could show pre-service students how to give up their need for perfect order in their classrooms. In a similar vein, Starley (2019) emphasized the role of the educational psychologist in developing coping strategies for teachers with maladaptive perfectionist behaviors.

The research studies presented above used different perfectionism measures, based on more or less different theoretical perspectives. In the present contribution, we present evidence regarding the psychometric properties of the PI (Hill et al., 2004), a questionnaire that combined the most influential theoretical perspectives on perfectionism. By analyzing the entire item pool of the PI, we provide evidence regarding its internal validity. Furthermore, we focused on schoolteachers because the educational environment encourages the achievement of high standards (Flett et al., 2009), where there is a strong expectancy for high performance (Schruder et al., 2014). Being a teacher involves job-specific responsibilities that are similar to various facets of perfectionism. These responsibilities include encouraging students to achieve higher standards (i.e., having high standards for others), organizing and planning each lesson in detail, having a high concern over mistakes (i.e., close self-monitoring in order to avoid teaching mistakes). Therefore, the perfectionistic tendencies described above (i.e., having high standards for students, organizing and planning each lesson, monitoring the mistakes made by students) “come with the job” in the case of teachers, and this could have a negative impact on the psychometric properties of the PI (Hill et al., 2004). Because these forms of perfectionism are job-related actions, teachers' responses to items corresponding to these scales will not reflect own personal options, but rather the degree to which the respondent is performant as a teacher. This could lead to large correlations between these scales, resulted from the fact that all these behaviors are required by the respondents' job.

## Materials and Methods

### Participants

We recruited 633 participants from public schools in Western regions of Romania, using a snowball sampling approach detailed in the Procedure sub-section. Most participants were female (81.18%), taught in primary schools (35.20%) and in secondary schools (45.66%), and were mostly from schools located in urban areas (55.92%). Their mean age was 42.11 years (SD = 9.80), and their mean tenure was 17.61 years (SD = 10.06). More details regarding the study sample are presented in Table 1.

**Table 1:** Descriptive statistics of the sample included in the study.

		**Age (years)**		**Teaching experience (years)**	
	***N***	**Mean**	**SD**	**Mean**	**SD**
*Total sample*	633	42.11	9.80	17.61	10.06
Male	106	41.75	11.18	13.98	9.58
Female	505	42.26	8.98	18.30	9.95
***School level***					
Primary school teachers	219	41.84	9.43	19.04	10.76
Secondary school teachers	284	41.97	9.57	16.28	9.63
High-school teachers	112	44.06	8.58	18.17	9.12
***Type of locality***					
Urban	354	42.79	9.23	18.13	9.95
Rural	230	41.69	9.69	17.02	10.19

*N = 633. Any differences between the cumulated number of teachers for each category and the declared sample size are due to existing non-responses in that particular category*.

### Measure

Perfectionism was assessed using the PI (Hill et al., 2004). The PI (Hill et al., 2004) has 59 items corresponding to 8 forms of perfectionism: *Organization* (sample item: “I like to always be organized and disciplined,”)*, High Standards for Others* (sample item: “I usually let people know when their work isn't up to my standards”)*, Striving for Excellence* (sample item: “My work needs to be perfect, in order for me to be satisfied”)*, Planfulness* (sample item: “I think through my options carefully before making a decision”), *Concern over Mistakes* (sample item: “If I make mistakes, people might think less of me”), *Need for Approval* (sample item: “I'm concerned with whether or not other people approve of my actions”), *Parental Pressure* (sample item: “I always felt that my parent(s) wanted me to be perfect”), and *Rumination* (sample item: “When I make an error, I generally can't stop thinking about it”). Respondents must rate their agreement with each item using a 5-point Likert scale (from *1—strongly disagree* to *5—strongly agree*). The PI (Hill et al., 2004) was translated from English into Romanian by a university English teacher. Later, for the correspondence of the meaning, the Romanian version was back-translated into English by another university English teacher. Finally, translators and researchers analyzed the translation process to ensure that the true meaning of the concepts was preserved after the translation process. The reliability indices (i.e., Cronbach's alpha) of the PI scales was good: *Organization* (α = 0.862)*, High Standards for Others* (α = 0.734)*, Striving for Excellence* (α = 0.811)*, Planfulness* (α = 0.740), *Concern over Mistakes* (α = 0.830), *Need for Approval* (α = 0.850), *Parental Pressure* (α = 0.906), and *Rumination* (α = 0.848).

### Procedure

The sample of teachers was selected using two ways: (i) with the support of the school management or (ii) with the help of a teacher that recruited our participants among his/her colleagues. The teacher, with the agreement of the school principal, asked colleagues if they would like to participate in the study. All teachers who accepted, first completed an informed consent form, according to the Ethic standards in research with human subjects. Both the consent form and the PI were administered in a paper-and-pencil format. The participants were not remunerated for participating in this study.

### Data Analyses

We conducted confirmatory factor analyses (CFA) using the *lavaan* package (Rosseel, 2012) in R. Firstly, we tested the eight-factor structure suggested by Hill et al. (2004). Secondly, we tested the two-factor solution theorized by Hill et al. (2004), which contains a factor named *Conscientious perfectionism* and a factor named *Self-evaluative perfectionism*. Initial investigations regarding the distribution of the responses to PI items indicated that the responses were not normally distributed (i.e., most Shapiro-Wilk tests were statistically significant). Therefore, we estimated our models using the *maximum likelihood* method, with robust standard errors (MLR). The MLR estimation implemented in *lavaan* allows for fitting models with non-normal distribution using Yuan-Bentler corrections for non-normal and missing data (Rosseel, 2012). Following the recommendations provided by Kenny et al. (2015), we computed the root mean square error of approximation (RMSEA) for the baseline model to check whether incremental fit indices (e.g., the comparative fit index, the incremental fit index, the Tucker-Lewis index) are informative in the case of our model. The RMSEA for the baseline model was 0.128, which is smaller than the threshold value of 0.158 suggested by Kenny et al. (2015) for considering the incremental indices. Therefore, we assessed model fit using the root mean square error of approximation (RMSEA, the acceptable fit is indicated by values below 0.08—Browne and Cudeck, 1993), and the standardized root mean square residual (SRMR—acceptable fit is indicated by values below 0.08—Hu and Bentler, 1999).

In addition to the confirmatory analyses, we also investigated the convergent and discriminant validity of the factor solutions. For the convergent validity, we used the criteria proposed by Anderson and Gerbing (1988): the factor loadings should be larger than 0.40, and the average variance extracted (AVE) for each factor should be above 0.50. To assess the divergent validity of each latent variable, we compared the squared root of its AVE with the correlation values between that latent variable and the other latent factors. The divergent validity is not supported if the correlation values are higher than the square root of the AVE (Chin, 1998).

## Results

### Confirmatory Factor Analyses

The fit indices of our CFAs (presented in Table 2) suggested that the eight-factors model had fit indices below the 0.08 threshold value [RMSEA = 0.055, 90% CI = (0.053–0.057); SRMR = 0.071]. On the other hand, the results of the two-factors model suggested that this perspective does not provide adequate fit [RMSEA = 0.082, 90% CI = (0.080–0.083); SRMR = 0.124]. Although it had acceptable fit indices, the eight-factors model (presented in Table 4) had some issues regarding the convergent and divergent validity of its factors. Firstly, although most factor loadings were larger than 0.40 (i.e., only the loadings of item 13 and item 3 did not reach this threshold), the average variance extracted by the eight-factors solution reached the 0.50 value only in the case of *Perceived Parental Pressure* factor (AVE = 0.59). The other AVE values suggested that the latent factors explained between 29% (the case of *High Standards for Others*) and 46% (the case of *Striving for Excellence*) of the variance of their items. This means that the eight-factors solution does not meet the criteria for convergent validity, as defined by Anderson and Gerbing (1988).

**Table 2:** Fit indices of the two alternative models.

**Model**	**Robust discrepancy**	**Robust RMSEA**		**Robust SRMR**
		**Value**	**90% CI**	
8-factor model	χ^2^(1567) = 4182.99, *p* < 0.001	0.055	[0.053–0.057]	0.071
2-factor model	χ^2^(1594) = 7458.66, *p* < 0.001	0.082	[0.080–0.083]	0.124
Additional model	χ^2^(1482) = 4606.48, *p* < 0.001	0.058	[0.056–0.060]	0.075

Secondly, the divergent validity of the eight factors was generally poor. The correlation matrix between the eight scales is presented in Table 3, and the squared value of the AVE index is included in the diagonal. The results presented in Table 3 suggested that divergent validity is problematic in the case of about half of the scales (i.e., *High Standards for Others, Concern over Mistakes, Need for Approval*, and *Rumination*). In the case of these scales, the squared value of the AVE is smaller than the correlation value between that scale and other factors included in the questionnaire. Simply put, these scales share more variance with other scales, than with own items. Furthermore, inter-factor correlation values are up to 0.95, which raised serious concerns regarding the divergent validity of these factors.

**Table 3:** Correlations matrix between the eight latent factors.

	**Org**	**StrExc**	**Plan**	**HSO**	**CoM**	**Nap**	**ParPr**	**Rum**
Org	*0.67*							
StrExc	0.40	*0.68*						
Plan	0.64	0.45	*0.55*					
HSO	0.16	0.58	0.26	*0.54*				
CoM	0.09	0.60	0.31	0.73	*0.63*			
Nap	0.07	0.56	0.26	0.78	0.95	*0.65*		
ParPr	0.06	0.51	0.17	0.44	0.55	0.48	*0.76*	
Rum	0.10	0.61	0.30	0.74	0.92	0.95	0.55	*0.67*

*N = 633. Squared AVE values are presented on the diagonal, in italics. Org, organization; StrExc, striving for excellence; Plan, planfulness; HSO, high standards for others; CoM, concern over mistakes; Nap, need for approval; ParPr, perceived parental pressure; Rum, rumination*.

### Additional Analyses

**Figure 1:**
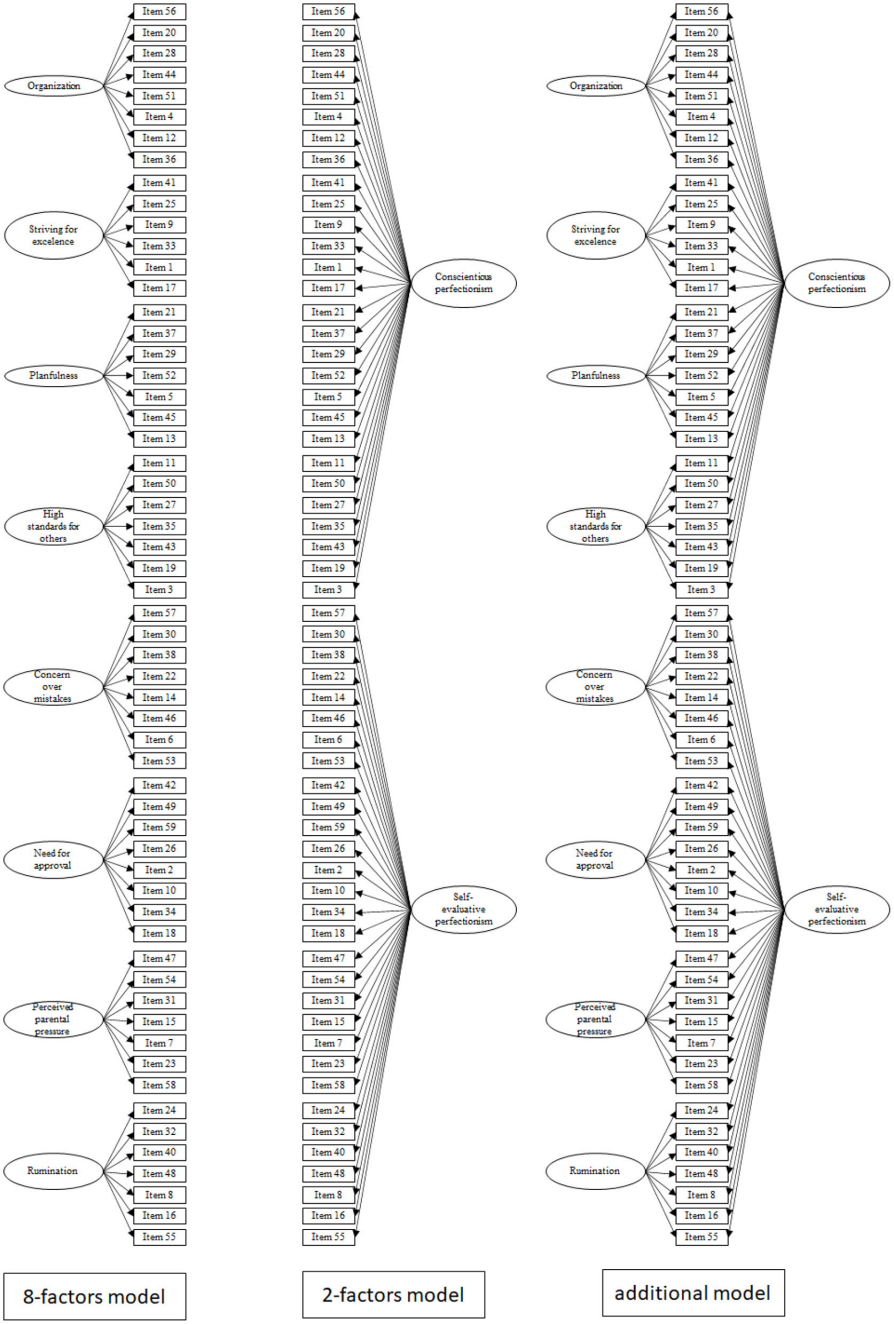
The three models tested.

Given the poor divergent validity of the scales, we concluded that the scales do not assess different psychological variables. Therefore, we conducted an additional analysis to investigate whether the items have specific variance on the latent variables defined by the eight-factors model, or on the latent variables defined by the two-factors model. In this analysis (i.e., a bifactor analysis), the variance of each item is distributed between the solutions (i.e., the eight-factors and the two-factors) that are tested simultaneously in an orthogonal model (see Figure 1 for a representation of the eight-factors, two-factors, and additional model). Consequently, the variance of each item is divided between a latent variable from the eight-factors solution and a latent variable from the two-factors solution. This analytical approach is superior to the traditional higher-order confirmatory factor analyses because it is more appropriate when it comes to dealing with multidimensionality issues (i.e., it leaves the possibility of having dimensions of the phenomenon that are independent of the general factor—Dunn and McCray, 2020), and it provides better fit of the dataset (Cucina and Byle, 2017).

To investigate how each latent variable accounts for the total variance of the items included in the analysis, we calculated the explained common variance (ECV). The ECV is computed as the sum of the squared loadings of those latent variables, divided by the sum of all squared loadings in the model. Therefore, the ECV can be interpreted as a percentage of variance accounted by a latent variables, out of the entire variance captured by that model.

Although this type of analysis (i.e., a bifactor analysis) usually contrasts a multi-factor solution with a one-factor solution, the existence of a one-factor solution is unlikely because many correlations presented in Table 2 also had values close to 0. Furthermore, a two-factor solution was also tested by previous studies (Hill et al., 2004; Cruce et al., 2012).

To ease their interpretation, the item loadings resulted from the additional analysis are also presented in Table 3, together with the loadings from the models that specified only the eight-factors model and only the two-factor model). A visual investigation of factor loadings presented in Table 4 revealed that item loadings had close to null values in the case of *Concern over Mistakes, Need for Approval*, and *Rumination*. This suggests that the variance of these items is not specific to latent variables from the 8-factors model, but to the *Self-evaluation* latent variable. Regarding the remaining five latent variables, most of their items had loadings above 0.40, which suggests that the latent variables are distinct enough to account for item variance. The ECV index suggested that the *Conscientiousness* and *Self-evaluation* accounted for 61% of all explained variance, while the other eight factors only accounted for 39%. On the one hand, this result is a strong argument for reconsidering the eight-factors solution. On the other hand, the *Conscientiousness* and *Self-evaluation* are not similar regarding their capacity to explain item variance. *Self-evaluation* accounts for about 40% of all item variance, while most of its subcomponents explain less than 5% of item variance (i.e., *Concern over mistakes* = 2%; *Need for approval* < 1%; *Rumination* = 2%), while *Perceived parental pressure* is the only subcomponent that still has specific variance (i.e., ECV = 0.12). The *Conscientiousness* latent variable accounts for 21% of the explained variance, while its sub-components explain 21% increment of the explained variance (*Organization* = 6%, *Striving for Excellence* = 7%, *Planfulness* = 4%, *High Standards for Others* = 7%). This means that the *Conscientiousness* sub-components can be differentiated and should not be integrated into a single, second-order factor.

**Table 4:** Standardized loadings of the CFA analyses.

	**8 factors model**								**2 factors model**		**Bifactor model**									
	**Org**	**StrExc**	**Plan**	**HSO**	**CoM**	**Nap**	**ParPr**	**Rum**	**Cs**	**SEv**	**Org**	**StrExc**	**Plan**	**HSO**	**CoM**	**Nap**	**ParPr**	**Rum**	**Cs**	**SEv**
it56	0.77								0.64		0.47								0.59	
it20	0.75								0.59		0.65								0.50	
it28	0.73								0.64		0.50								0.56	
it44	0.67								0.62		0.24								0.64	
it51	0.67								0.56		0.37								0.53	
it4	0.61								0.49		0.56								0.38	
it12	0.58								0.65		−0.01								0.74	
it36	0.58								0.56		0.17								0.57	
it41		0.78							0.51			0.71							0.37	
it25		0.73							0.41			0.62							0.39	
it9		0.72							0.41			0.56							0.44	
it33		0.65							0.50			0.56							0.30	
it1		0.56							0.59			0.45							0.35	
it17		0.42							0.50			0.09							0.60	
it21			0.66						0.51				0.50						0.46	
it37			0.65						0.49				0.44						0.46	
it29			0.61						0.47				0.50						0.40	
it52			0.54						0.43				0.35						0.42	
it5			0.53						0.46				0.31						0.43	
it45			0.49						0.40				0.32						0.36	
it13			0.39						0.43				0.00						0.47	
it11				0.64					0.27					0.62					0.21	
it50				0.62					0.18					0.55					0.08	
it27				0.59					0.20					0.63					0.11	
it35				0.57					0.39					0.48					0.37	
it43				0.51					0.29					0.48					0.24	
it19				0.45					0.26					0.44					0.21	
it3				0.32					0.27					0.28					0.22	
it57					0.72					0.68					0.22					0.68
it30					0.71					0.68					0.06					0.69
it38					0.66					0.64					0.28					0.63
it22					0.64					0.62					0.01					0.64
it14					0.63					0.62					−0.12					0.64
it46					0.62					0.60					0.44					0.58
it6					0.53					0.50					0.05					0.52
it53					0.40					0.37					0.42					0.35
it42						0.76				0.71						0.02				0.72
it49						0.72				0.69						0.01				0.71
it59						0.72				0.69						0.02				0.69
it26						0.64				0.64						0.02				0.64
it2						0.61				0.57						0.01				0.60
it10						0.60				0.59						0.00				0.60
it34						0.58				0.55						0.00				0.57
it18						0.55				0.52						0.01				0.55
it47							0.87			0.58							0.71			0.48
it54							0.86			0.59							0.70			0.49
it31							0.79			0.48							0.71			0.37
it15							0.74			0.42							0.70			0.31
it7							0.72			0.53							0.55			0.47
it23							0.72			0.56							0.54			0.49
it58							0.65			0.39							0.60			0.28
it24								0.72		0.70								0.16		0.66
it32								0.71		0.67								0.51		0.66
it40								0.71		0.67								0.40		0.69
it48								0.70		0.68								0.07		0.50
it8								0.66		0.66								0.00		0.64
it16								0.66		0.63								0.09		0.70
it55								0.52		0.52								0.06		0.66
AVE	0.45	0.46	0.31	0.29	0.40	0.42	0.59	0.45	0.21	0.36	–	–	–	–	–	–	–	–	–	–
ECV	0.15	0.11	0.09	0.08	0.13	0.14	0.17	0.13	0.37	0.63	0.06	0.07	0.04	0.07	0.02	<0.01	0.12	0.02	0.21	0.40

*N = 633. Org, organization; StrExc, striving for excellence; Plan, planfulness; HSO, high standards for others; CoM, concern over mistakes; Nap, need for approval; ParPr, perceived parental pressure; Rum, rumination; Cs, conscientious perfectionism; SEv, slf-evaluative perfectionism*.

## Discussion

In the present research study, we investigated the internal validity of the PI (Hill et al., 2004) in an occupation that encourages perfectionistic tendencies in own behavior or in students' behavior (Shim et al., 2020). Our focus on teacher perfectionism was motivated by the fact that previous studies reported that it is a powerful predictor for teacher efficiency and teacher burnout (Craioveanu, 2014; Ghorbanzadeh and Rezaie, 2016), and can have an impact on students' variables (Lozano et al., 2019). We collected data from a large sample of schoolteachers, and we analyzed the factor structure of the PI (Hill et al., 2004) using confirmatory factor analyses.

Our CFA results suggested that the initial, eight-factor structure of TPI provided a reasonable fit on our sample of teachers. This result was encouraging because Hill et al. (2004) did not conduct a factor analysis (confirmatory or exploratory) on the entire set of items. However, additional analyses revealed that most of the latent factors explained suboptimal percentages of item variance (i.e., values below 50%), which suggested that most of the item variance remained unexplained by the eight-factor solution. Furthermore, we found evidence for problematic divergent validity in the case of about half of the scales. Although strong between-scale correlations were also present in the original study (Hill et al., 2004), the median correlation value was larger in our study (i.e., *r* = 0.49), as compared with the original study (*r* = 0.37). Based on these findings, we concluded that the eight-factor solution had serious psychometrics limitations regarding convergent and divergent validity, and we conducted additional investigations.

The original model, Hill et al. (2004) theoretized that specific factors are not independent from the general factors. However, the bifactor analysis addresses some practical issues regarding the divergent validity of the specific factors. These issues were not initially anticipated by the theoretical framework developed by Hill et al. (2004), and neither by the empirical evidence that they presented (i.e., their factor analyses based on scale scores). The bifactor analyses indicated that 61% of all explained variance can be attributed to the factors suggested by Hill et al. (2004): *Conscientiousness* and *Self-evaluative perfectionism*. However, the two factors had rather different roles. On the one hand, *Self-evaluative perfectionism* accounted for most of the explained variance (40% of the total variance), while its sub-dimensions (i.e., *Need for Approval, Rumination, Concern over Mistakes*) had very weak relations with own items. This suggests that these sub-dimensions do not have specific variance and their scores do not capture different forms of perfectionism. Previous studies reported that socially-prescribed perfectionism (e.g., high need for approval or high concern over mistakes) is related to experiencing self-conscious emotions such as shame, guilt and embarrassment (Tangney, 2002). Because these forms of perfectionism were not differentiated on our teacher sample, the PI (Hill et al., 2004) has limited capabilities regarding the differential diagnostic of the perfectionist tendencies that could explain psychological strain. However, because confirmatory analyses on the entire set of PI items are scarce, it is premature to conclude that the components of *Self-evaluative perfectionism* are generally indistinguishable one from another. For example, results suggested that the *Perceived Parental Pressure* captures specific variance that is distinguishable from its super-ordinate factor (i.e., *Self-evaluative perfectionism*). Therefore, it seems that this scale has good discriminant validity and could be seen as a form of perfectionism that is separated from the super-ordinate factors. This result can be explained by the fact that *Perceived Parental Pressure* can be interpreted as an antecedent to perfectionism (Stricker et al., 2019). To conclude, future studies should provide additional evidence regarding the specificity of the scales that compose these two forms of perfectionism. On the other hand, the *Conscientious perfectionism* supra-factor had a different role. In this case, the explained variance was equally distributed between *Conscientious perfectionism* (that accounted for 21% of the total explained variance) and its sub-scales (i.e., *Organization, Striving for Excellence, Planfulness, High Standards for Others*—that together accounted for 24% of the explained variance). This result suggests that the four scales assess different forms of perfectionism, each with its unique variance.

From a teacher assessment perspective, our results suggested that *Self-evaluative perfectionism* could be used as a single composite score, while component (or scale) scores should be used in the case of *Conscientious perfectionism*. This is important because the two forms of perfectionism also have different functionalities. On the one hand, the *Self-evaluative perfectionism* is associated with low levels of trait emotional stability (i.e., trait neuroticism—Cruce et al., 2012), while *Conscientious perfectionism* is associated with trait conscientiousness (Cruce et al., 2012). Previous research studies suggested that teacher neuroticism is associated with low students' self-efficacy, while teachers' conscientiousness was a predictor for the students' reports of support from the teacher (Kim et al., 2018). Based on these findings, future studies should investigate whether different forms of teacher perfectionism (i.e., self-evaluative or conscientious perfectionism) are associated with students' variables. Furthermore, the *Conscientious perfectionism* scales could be linked with individual differences in structuring and conducting teaching activities. For example, Decker and Rimm-Kaufman (2008) reported that trait conscientiousness was significantly associated with the schoolteachers' focus on the teaching process. According to their results, highly conscientious schoolteachers believe that classroom activities should have a set of explicit rules that need to be reinforced constantly, that they should organize and discuss the schedule of the day with their students, and that the teachers' primary goal is to establish and maintain classroom control (Decker and Rimm-Kaufman, 2008). Based on the relations presented above, it is possible that different forms of conscientiousness perfectionism could be related to different teacher beliefs regarding the instructional process. In this vein, future studies could investigate the relations between the PI scales (Hill et al., 2004) and various models that describe teachers' beliefs regarding the instructional process (Decker and Rimm-Kaufman, 2008), or their approaches to teaching (Trigwell and Prosser, 2004). For example, the assessment of teachers' beliefs regarding the instructional process include items that refer to scheduling the school day, establishing a morning routine in the classroom, or reinforcing the rules for students' classroom behavior (Decker and Rimm-Kaufman, 2008). Future studies could investigate whether the endorsement of the teachers' beliefs mentioned earlier is associated with teachers' forms of conscientious perfectionism.

## Limitations

The present research study has some limitations that should be acknowledged. Firstly, our sample was unbalanced in terms of participants' gender (i.e., about 80% of the participants were female) and the school level (i.e., only 18% of the participants were high school teachers). On the one hand, previous research on Romanian samples did not yield gender differences regarding the levels of perfectionism (Macsinga and Dobriţa, 2010). On the other hand, the gender differences regarding some components related to Self-evaluative perfectionism (e.g., rumination—Johnson and Whisman, 2013) are very well documented in the literature. However, given the gender imbalance present in the schoolteacher population, a gender-balanced sample was difficult to attain. Regarding the school level, it is possible that high school teachers approach teaching in a different manner, as compared with primary or secondary school teachers. Therefore, their responses to some items (e.g., High standards for others) could have been different. Finally, future research studies should extend this investigation by including other multidimensional perfectionism scales (e.g., Frost et al., 1990; Hewitt and Flett, 1991) and external criteria relevant for the educational environment (e.g., approaches to teaching—Trigwell and Prosser, 2004).

## Conclusions

In the present paper, we investigated the factor structure of a comprehensive inventory of perfectionism scales (i.e., the PI—Hill et al., 2004) on a large teacher sample. We found that teachers provided differentiated responses to the items of conscientious perfectionism scales, not to the items of the self-evaluative perfectionism scales. This suggests that the PI (Hill et al., 2004) could be useful to investigate how perfectionism is related to various teaching behaviors linked to conscientiousness, but the PI could be a limited measure in explaining teacher strain and teacher unwell-being.

## Data Availability Statement

The raw data supporting the conclusions of this article will be made available by the authors, without undue reservation.

## Ethics Statement

Ethical review and approval was not required for the study on human participants in accordance with the local legislation and institutional requirements. The patients/participants provided their written informed consent to participate in this study.

## Author Contributions

ES selected the topic, organized the collection of the data, and contributed in the writing of the manuscript. LM contributed to the design of the study, performed the statistical analyses, and contributed in the writing of the manuscript. Both authors contributed to the manuscript revision and approved the submitted version.

## Conflict of Interest

The authors declare that the research was conducted in the absence of any commercial or financial relationships that could be construed as a potential conflict of interest.
